# Copy number variation is associated with gene expression change in archaea

**DOI:** 10.1099/mgen.0.000210

**Published:** 2018-08-24

**Authors:** Keely A. Dulmage, Cynthia L. Darnell, Angie Vreugdenhil, Amy K. Schmid

**Affiliations:** ^1^​ University Program in Genetics and Genomics, Duke University, Durham, NC, USA; ^2^​ Biology Department, Duke University, Durham, NC, USA; ^3^​ Center for Genomics and Computational Biology, Duke University, Durham, NC 27708, USA

**Keywords:** archaea, genomic plasticity, copy number variation, computational genomics

## Abstract

Genomic instability, although frequently deleterious, is also an important mechanism for microbial adaptation to environmental change. Although widely studied in bacteria, in archaea the effect of genomic instability on organism phenotypes and fitness remains unclear. Here we use DNA segmentation methods to detect and quantify genome-wide copy number variation (CNV) in large compendia of high-throughput datasets in a model archaeal species, *Halobacterium salinarum*. CNV hotspots were identified throughout the genome. Some hotspots were strongly associated with changes in gene expression, suggesting a mechanism for phenotypic innovation. In contrast, CNV hotspots in other genomic loci left expression unchanged, suggesting buffering of certain phenotypes. The correspondence of CNVs with gene expression was validated with strain- and condition-matched transcriptomics and DNA quantification experiments at specific loci. Significant correlation of CNV hotspot locations with the positions of known insertion sequence (IS) elements suggested a mechanism for generating genomic instability. Given the efficient recombination capabilities in *H. salinarum* despite stability at the single nucleotide level, these results suggest that genomic plasticity mediated by IS element activity can provide a source of phenotypic innovation in extreme environments.

## Data Summary

Gene expression and ChIP raw and normalized microarray data are available in the Duke Digital Repository at accession DOI: 10.7924/r4pz54w7h (direct link https://dx.doi.org/10.7924/r4pz54w7h). A subset of these data that were previously published is publicly available at the GEO accessions listed in Table S1 (available in the online version of this article). RNA-seq data are available at SRA accession number SRP108734 and in Table S6. All computer code is available at https://github.com/amyschmid/Halobacterium_CNV and Duke Digital Repository at the accession listed above.

**Impact Statement**

Microbial genomes are frequently rearranged, leading to discontinuity in the DNA of strains of the same species. Although once thought to be harmful to bacterial viability, recent evidence points to the benefits of such rearrangements to bacteria, enabling new phenotypes such as antimicrobial resistance. Although widely studied in bacteria, genome-wide structural variation has not yet been extensively investigated in archaea. In this study, the causes and phenotypic effects of genomic plasticity were quantified in an archaeal model species that lives in saturated salt lakes. We applied a genomic segmentation method to detect new hotspots for genomic changes in a compendium of nearly 3 million data points for a model archaeal species. Many of these genomic changes result in up- or down-regulation of genes. Mobile genetic elements are frequently found near these hotspots, suggesting a mechanism for generating genomic instability. As whole genome sequencing and transcriptomics data sets grow in the microbial genomics research community, we hope that our method will be useful for analysis in a wide range of microbial species, representing an important method to differentiate between gene expression changes due to regulation or due to changes in genomic structure. Overall, this study identifies a frequently used strategy for innovating new stress resistance phenotypes in extreme environments.

## Introduction

Microbes remain viable in the face of a stressful environment using a multitude of mechanisms. Genomic plasticity, once thought to result only in deleterious mutations, is now recognized to enable rapid generation of biodiversity through changes in the abundance of certain genes and lead to new regulatory programmes [1]. Events contributing to genomic structural changes include rearrangements, DNA copy number variations (amplifications or deletions), inversions and translocations [1, 2]. Such rearrangements may occur on a kilobase or megabase scale. Genetic rearrangements are common in organisms from all three domains of life and are frequently mediated by illegitimate homologous recombination at myriad types of interspersed, mobile repetitive genomic elements [1, 3, 4]. In bacteria and archaea, the most frequent ‘mobilome’ elements are insertion sequence (IS) elements, which are typically ~0.5–2 kb in length, encode a transposase, and are flanked by terminal inverted repeats [5]. Various families of IS elements are widely distributed across species and abundant within species [3, 4, 6–8]. In bacteria, although most IS-mediated rearrangements are neutral or deleterious, some lead to beneficial phenotypes such as antibiotic resistance [1, 9], stress resistance [2, 10], or increased virulence [11], suggesting a unique source of adaptive innovation. Active IS elements are also known to facilitate genomic rearrangement in archaea, although the phenotypic effects remain unclear [5, 12–14].

Hypersaline-adapted species of archaea have long been used as model systems for investigation of genomic plasticity in micro-organisms. For example, in the model organism *Halobacterium salinarum* strain R1, frequent transposition of IS elements in the promoter or coding regions of the plasmid-encoded gas vesicle gene cluster disrupts the formation of these organelles, resulting in a phenotypic change from opaque to clear colonies at rates as high as 10^–2^ [14, 15]. The genomes of halophilic archaea are also highly polyploid, with some species containing over 20 copies per cell during rapid growth [16]. Polyploidy provides templates for efficient DNA repair, enabling survival in irradiated salt flat environments [17]. Polyploidy can also provide another potential source of phenotypic variation by maintaining heterozygosity in the presence of selection [18].

The instability of large extrachromosomal megaplasmid genetic elements has been well documented in NRC-1 and R1 strains of *H. salinarum*, including segmental inversions, duplications and deletions [19–21]. The chromosomes of *H. salinarum* strains, like those of other archaea, are gene-dense with polycistronic operons and monocistronic transcripts found on both strands [22]. NRC-1 has one large chromosome (~2 Mb) and two smaller megaplasmids, pNRC100 and pNRC200 (191 and 365 kb, respectively). These megaplasmids contain several large repeat regions, with ~120 kb shared in common between the two [23]. In contrast, *H. salinarum* strain R1 harbours four megaplasmids that differ structurally from NRC-1, with topological rearrangements, deletions and duplications of several regions compared to the two NRC-1 megaplasmids [21]. Surprisingly, however, outside of these genetic rearrangements, very few SNPs were observed between the genome sequences of the two strains despite decades of divergent history [21]. The background mutation rate of halophilic archaea has been estimated to be nearly one order of magnitude lower than that of mesophilic species [24].

Together these data suggest that rearrangement by IS elements, efficient recombination systems and polyploid genomes may be important generators of biodiversity in this model archaeal species. However, the frequency of instability has not yet been systematically investigated genome-wide in *H. salinarum* strains or in archaea generally. Whether and how such genomic plasticity influences phenotypes such as gene expression also remain unclear.

Here we expand the understanding of *H. salinarum* genomic plasticity by investigating the link between instability and gene expression in strain NRC-1, employing computational methods to detect large (kilobase scale) duplication and deletion events in large compendia of microarray data. These events, often referred to as copy number variants (CNVs), have long been the subject of intense interest in the field of human genetic variation [25, 26]. Segmentation methods are frequently used to observe CNVs in array comparative genomic hybridization (aCGH) data [27]. By applying a chromosome-segmenting algorithm designed to detect DNA CNVs to the analysis of 1154 mRNA gene expression microarray datasets for *H. salinarum*, we detected the co-expression of large co-linear regions of the megaplasmids and main chromosome, each spanning multiple operons. Meta-analysis of microarray data for control genomic DNA reveals CNV hotspots, some of which correspond to co-expressed gene regions, suggesting phenotypic consequences for CNVs. In validation experiments, we identify specific DNA amplification and deletion events and link these directly to changes in gene expression. CNV hotspot regions are significantly associated with flanking IS elements, suggesting a mechanism for varying gene dosage. The phenotypic and fitness consequences of these rearrangements in *H. salinarum* are discussed. The computational methods applied here are useful for any microbial system for which gene expression and DNA quantification data exist.

## Methods

### Strains and growth conditions

All strains used here were derived from *Halobacterium* sp. NRC-1 (ATCC 700922). Empty vector control strain *Δura3/pMTFCHA* (KAD101) and histone overexpression strain *Δura3/pMTFCHA :: hpyA* (KAD102) were originally described by Dulmage *et al*. [28]. Mutant strain *Δura3Δhlx2* (hereafter, ∆*hlx2*) was originally described by Darnell *et al*. [29]. Isogenic parent control strain NRC-1 *Δura3* was described by Peck *et al*. [30]. A complete strain list is given in Table S2. Strains were maintained to avoid accumulation of CNVs. Each strain was first streaked onto plates from frozen storage at −80 °C, with no re-streaking once grown on plates. For routine culturing, single colonies were first inoculated into 5 ml liquid starter cultures and grown to early stationary phase to synchronize growth phase (OD_600_ of ~1) at 42 °C with 225 r.p.m. agitation in ambient light in complex medium (CM; per litre: 250 g NaCl, 20 g MgSO_4_· 7H_2_O_,_ 3 g sodium citrate, 2 g KCl, 10 g peptone). Cultures were diluted to an OD_600_ of ~0.05 into 50 ml cultures in CM and grown to mid-logarithmic phase for the final experiment (or stationary phase in some prior microarray experiments, Table S1). The growth medium was supplemented with uracil (50 µg ml^−1^) for growth of ∆*ura3* and ∆*hlx2* to complement the auxotrophy. CM was supplemented with the antibiotic mevinolin (1 µg ml^−1^) to maintain plasmids during growth of strains *KAD101* and *KAD102*.

### Concatenation and normalization of 1154 gene expression arrays

Raw data from 2308 existing gene expression microarrays for *H. salinarum* was normalized as follows. For each array, probes were identified that had a mean low-intensity scan value >0 and an unsaturated mean high-intensity scan value. Arrays with ≥1000 probes fitting this criterion, with an overall *R*^2^≥0.95 between the high- and low-intensity signals for given probes, were included in subsequent analyses. A linear regression model was then used to project the raw low-intensity values to raw high-intensity values in the instances where the mean high-intensity value reached saturation. Resultant data files were read and processed using the R software package *limma* from Bioconductor [31, 32]. Background subtraction and within-array normalization of probe intensities was performed as described by Dulmage *et al*. [28]. The expression value for each gene per array was then defined as the median probe value for that gene. The expression ratio for each gene per experiment was then calculated as the experimental condition divided by the wild-type control sample. The average of the dye-swap experiments was taken as the final value for each gene within each experiment. Quantile normalization was used to standardize the expression ratios across all of the arrays. The expression ratios across all experiments were then mean-centred and converted to z-scores, where final values correspond to distance from the mean in units of standard deviation.

These data totalled 1154 arrays when corresponding dye-swap control arrays were incorporated. A summary of GEO accession numbers and original publication references for these data are listed in Table S1. All quantile normalized data and corresponding detailed metadata are given in Table S3. Raw data, quantile normalized data and corresponding custom Python scripts used to normalize the raw data are freely accessible through the Duke Digital Repository at accession DOI: 10.7924/r4pz54w7h (direct link https://dx.doi.org/10.7924/r4pz54w7h).

### Detection and mapping of correlated regions of gene expression in normalized data from 1154 arrays

**Fig. 1.**
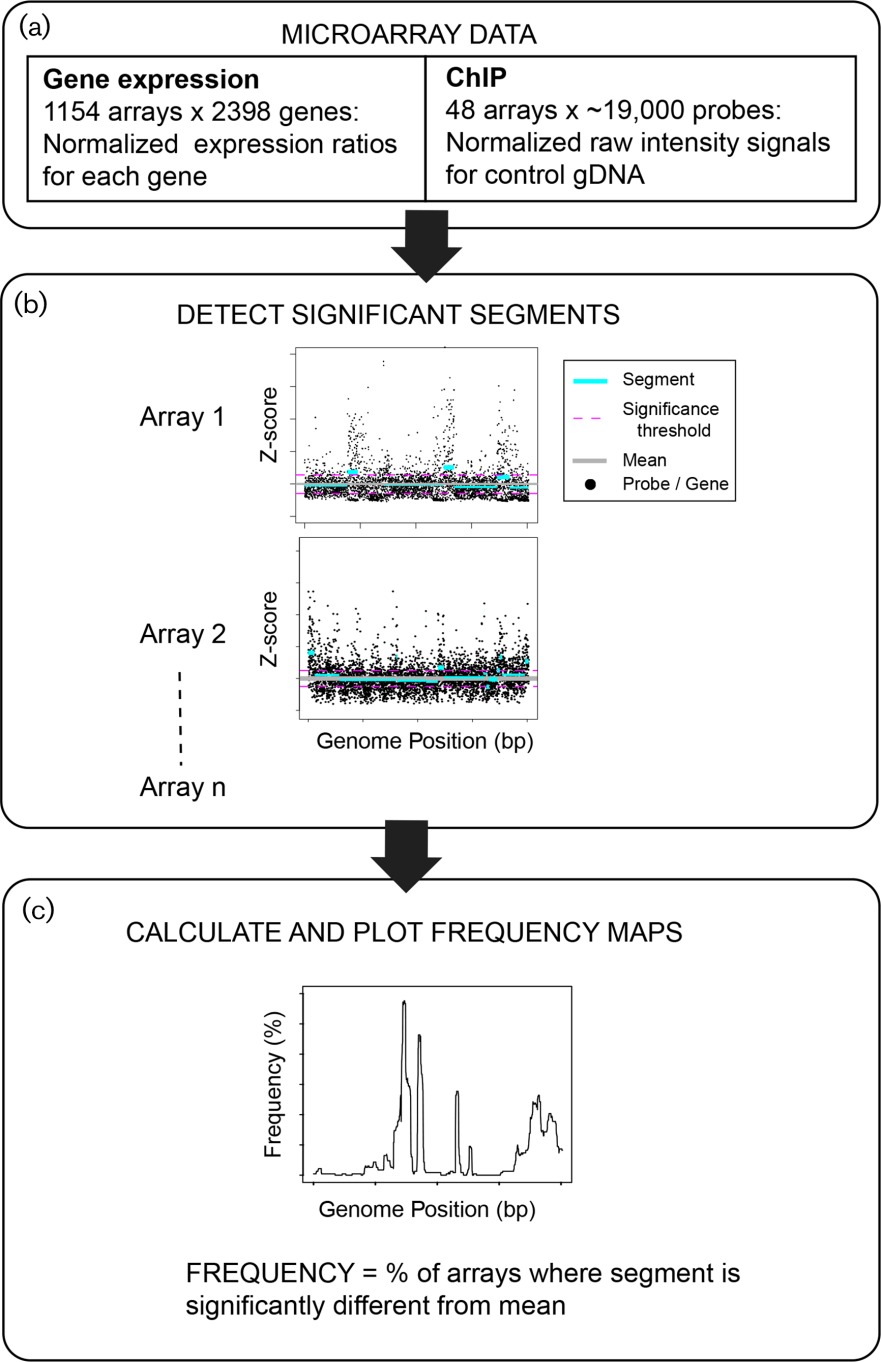
Microarray processing and workflow of chromosomal breakpoint analysis. The workflow consisted of (a) normalization of ChIP-chip and transcriptomic microarray data; (b) detection of correlated regions (segments) that met size and significance thresholds (see also Methods); and (c) plotting the composite segment frequency by genomic position across arrays. Panel (b) shows two examples of segmenting algorithm output for individual arrays. Individual points and lines are as in the key. As an example, ChIP-chip control data for the TrmB transcription factor grown in 0.1 % glucose is shown (array metadata, Table S1).

**Fig. 2.**
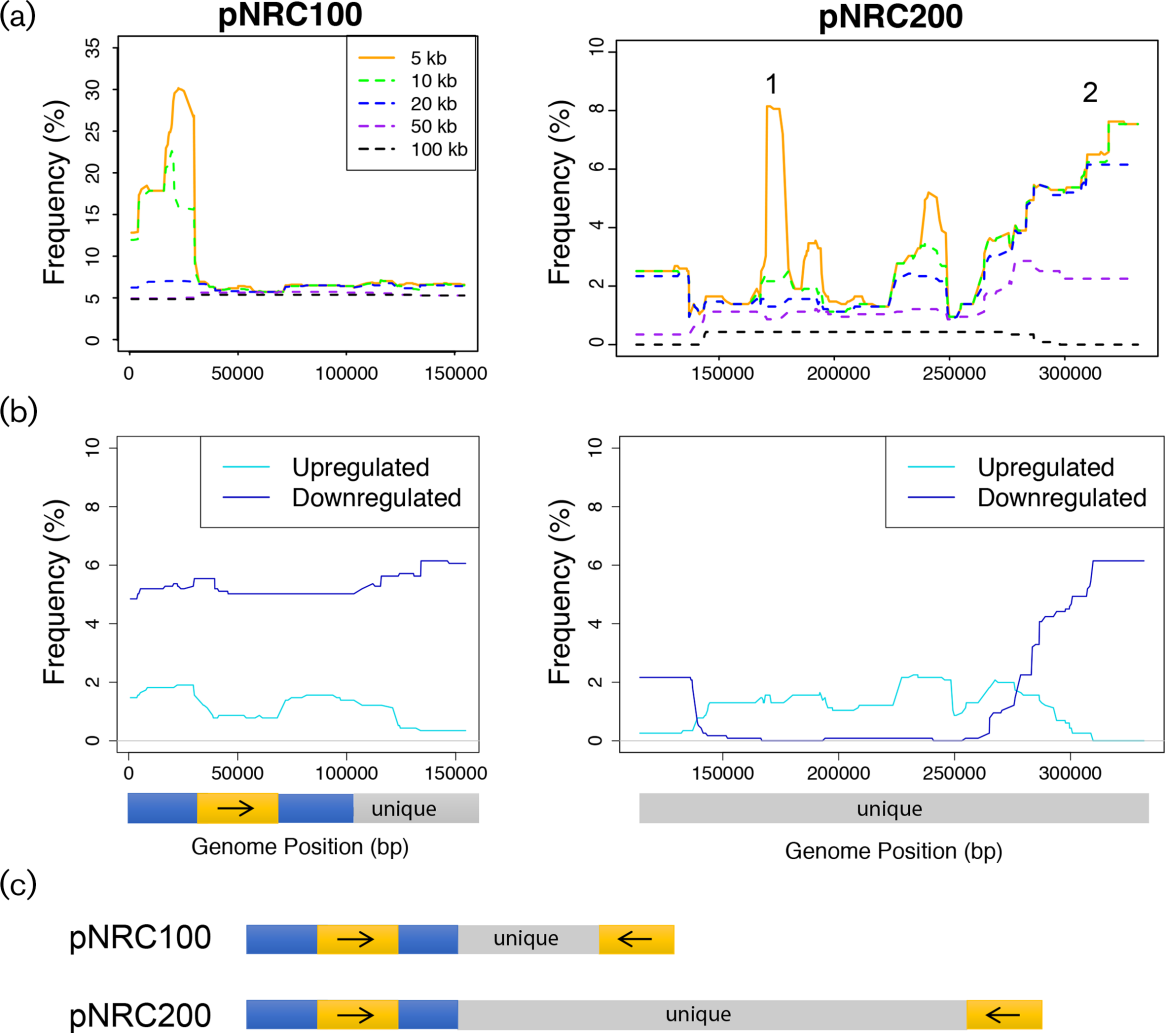
Regions of correlated gene expression in the megaplasmids of *H. salinarum*. (a) The frequency of gene expression segments meeting the minimum size and significance criteria (see key) is plotted against megaplasmid DNA coordinates. pNRC100 is shown at left and pNRC200 at right. Numbered peaks in the pNRC200 plot correspond to those discussed in the main text. (b) Directional regulation of correlated gene expression regions that are at least 20 kb in size. Bars under pNRC100 and pNRC200 represent the locations of repeat and unique regions as represented in the gene expression arrays [23]. (c) Diagram of direct repeat regions (dark blue), inverted repeat regions (orange) and unique regions (grey) in the megaplasmid sequences [23].

Probes with missing values in any experiment were removed prior to analysis. In the gene expression dataset, all experimental RNA samples were hybridized against a common mid-logarithmic phase standard reference RNA, and only those segments 1 standard deviation from the mean (i.e. meeting threshold), representing gene expression changes from this reference sample, were included in the resultant frequency maps. Normalized arrays were analysed and segmented using the R package DNAcopy as described by the authors, using default smoothing and segmentation parameters [27]. Briefly, DNAcopy uses a circular binary segmentation (CBS) algorithm to identify DNA regions from the genomic background in terms of copy number [27]. Specifically, correlated segments of gene expression, as determined by CBS, were subject to a z-score threshold of at least 1. Because the first 113 kb of pNRC100 and pNRC200 are identical, representing a large duplicated region, the first five genes assigned to pNRC200 on the gene expression arrays were already represented in the pNRC100 probes and thus were removed prior to mapping. Gene expression frequency maps (see Figs 2 and 4) were generated by calculating the number of times a probe was detected in segments meeting significance and size thresholds and then dividing this number by the total number of arrays in the analysis set (Fig. 1, Table S4).

To determine the significance of enrichment in gene functional categories within each large frequency peak (Figs 2 and 4), the start codon of each gene in the genome was located within those fragments meeting threshold criteria in the segmentation data (Table S4). Over-representation in archaeal clusters of orthologous genes (arCOG; [33]) functional categories for genes within segments differentially expressed in ≥5 % of the 1154 arrays was calculated using the hypergeometric test with Benjamini–Hochberg correction for multiple hypothesis testing ([28, 34, 35]; https://github.com/amyschmid/histone_arCOG).

### Detection of CNVs in chromatin immunoprecipitation (ChIP) microarray data

Raw intensity values were extracted from ChIP microarray experiments representing randomly sheared genomic DNA from the whole-cell extract control (also known as ‘input DNA’). Flagged array spots were removed from analysis and the remaining raw intensities were converted to z-scores. These values were then median normalized so that the majority of DNA ratios centred on zero. A total of 48 microarrays tiled at 500 bp resolution across the *H. salinarum* genome were analysed. These data were analysed using the DNAcopy segmentation algorithm [27] as described above. For generation of the composite frequency map, segments were filtered for those fragments that were at least 0.5 standard deviations from the mean and at least 5 kb in span (Table S5). As these arrays were not designed for the investigation of linearly arranged probes, the large duplicated regions on the megaplasmids were condensed into a single region regardless of their orientation within the genome or relative copy number, and thus we did not analyse data from the megaplasmids. Data from biological replicate experiments were kept separate.

GEO accession numbers, publication references and metadata for these experiments can be found in Table S1. All raw data, median normalized data and corresponding custom R scripts used to normalize the raw data are freely accessible through the Duke Digital Repository at accession DOI: 10.7924/r4pz54w7h (direct link https://dx.doi.org/10.7924/r4pz54w7h).

### Gene expression microarrays and analysis for strains KAD101 and KAD102

Three biological replicate cultures of strains *Δura3/pMTFCHA* (KAD101) and *Δura3/pMTFCHA :: hpyA* (KAD102) were grown to mid-logarithmic phase in CM supplemented with 50 µg uracil ml^−1^ and 1 µg mevinolin ml^−1^, then sub-cultured to an OD_600_ of 0.05 for further growth prior to harvesting at mid-logarithmic (OD_600_ of ~0.4) and stationary phases (OD_600_ of ~1.2). RNA extraction, quality control, labelling and hybridization to custom Agilent ORF arrays (six probes per gene) were performed as described previously [36]. Absence of contaminating DNA was verified by PCR. Each RNA sample was labelled and hybridized against *H. salinarum* NRC-1 wild-type grown under standard conditions (CM to OD_600_ of ~0.4 at 37 °C with shaking at 225 r.p.m.; [37]). Dye swaps were performed for each biological replicate. A total of 36 replicate data points were collected per gene in each sample. Spot ratios were determined using Agilent Feature Extraction and all further analysis was performed in the R statistical computing environment. Ratios were normalized within and between arrays using the R package *limma* [31, 38] in a pipeline adapted from Sharma *et al*. [36]. Significant differential gene expression between strains KAD101 and KAD102 was detected in resultant normalized gene expression data by Student’s *t*-test in the TM4 Multiple Experiment Viewer software (*P*<0.05) [39], then corrected for multiple hypothesis testing using Benjamini–Hochberg correction [35] in the R statistical environment. All raw and normalized microarray data are included in the Duke Data Repository for the full 1154 array dataset described above (https://dx.doi.org/10.7924/r4pz54w7h) and in Table S3.

### RNA-seq experiments and data processing

Triplicate starter cultures of Δ*ura3* and Δ*hlx2* were grown until stationary phase and then subcultured in CM supplemented with uracil (50 µg ml^−1^). At mid-logarithmic phase (OD_600_ of ~0.35–0.4), samples were collected, pelleted and stored at −80 °C. RNA was harvested and quality-checked as described by Sharma *et al*. [36]. Ribosomal RNA was removed using the Ribo-Zero rRNA Removal kit for bacteria (Illumina) as per the manufacturer's instructions and removal was verified using the Agilent Bioanalyzer RNA Nano 6000 chip. Libraries were prepared using a Stranded RNA-Seq Kit (KAPA) and TruSeq adapters (Illumina) as per the manufacturer's instructions. cDNA library quality was assessed by Bioanalyzer using a High Sensitivity DNA chip (Agilent). Samples were pooled and run in a single lane on an Illumina HiSeq 2500 device (Duke Sequencing and Genomics Technologies core). Reads of 50 bp were assessed for quality using FastQC [40] and adapter sequences were trimmed using TrimGalore! [41] and Cutadapt [42]. Resultant sequences were aligned to the *H. salinarum* NRC-1 reference genome (RefSeq: NC_002607.1, NC_002608.1, NC_001869.1) [23] using Bowtie2 [43]. SAM files were converted to BAM files and sorted using samtools [44]. Reads were assigned to genes and read counts were quantified using Python package HTSeq [45]. Raw and normalized data have been deposited in the NCBI GEO database at accession number GSE99730, in the Sequence Read Archive (SRA) accession number SRP108734 and in Table S6. Sequencing platform details are available at GEO accession GPL23553.

### Quantitative PCR detection of chromosomal instability

Genomic DNA was harvested from three biological replicate stationary phase (OD_600_ of ~1.0) cultures of KAD101 (*ura3/pMTFCHA*) and KAD102 (*ura3/pMTFCHA::hpyA*), and six biological replicate cultures of Δ*ura3* and Δ*ura3*Δ*hlx2.* Briefly, 1 ml of each culture was centrifuged at 2389 x ***g*** for 30 s and lysed by resuspension in 500 µl Tris-EDTA (TE) buffer (*H. salinarum* is an obligate halophile and lyses readily in low-salinity solutions). DNA lysates were homogenized by passage through a needle and clarified by 5 min centrifugation at 2389 x ***g***. RNA was removed by a 5 min room-temperature treatment with 250 µg RNAse A. Protein was digested with Proteinase K at 37 °C for 10 min. Samples were extracted once in an equal volume of phenol/chloroform/ isoamyl alcohol (25 : 24 : 1) and DNA was precipitated in ethanol and resuspended in TE buffer. Three 10-fold serial dilutions of 25 ng DNA were amplified using the SsoAdvanced SYBR Green Supermix (Bio-Rad) according to the manufacturer’s instructions. At least three technical replicates were analysed for each biological replicate. To detect amplified genomic regions in strain KAD102, DNA dosage was calculated relative to the ∆*ura3* parent strain and a control region using the ΔΔ*C*_t_ method [46, 47]. Specifically, the *VNG5097H* and *VNG5102H* ORFs were compared to the reference locus *VNG5019G*. These loci are contained within a region duplicated elsewhere on pNRC100 (pNRC100 : 1–112972) and so are compared to one another directly to assess differential DNA copy number. *VNG5148H* was compared to the reference locus *VNG5192H* (these genes are both within a region not previously known to be duplicated). For the deletion event in the *VNG0989C* ORF, raw *C*_t_ values from amplification of 250 pg of DNA using primers annealing to the region of *VNG0989C* were compared relative to negative controls: (a) primers annealing to reference locus *VNG1756G*; (b) *Escherichia coli* DH5α chromosomal DNA template with *VNG0989C* primers; and (c) water template with *VNG0989C* primers. Primers for all quantitative PCR (qPCR) experiments are listed in Table S2.

### Statistical analysis of IS element association with CNVs

CNV peak regions were defined as those genomic coordinates in the main chromosome in which 10 % or more of the arrays contained a fragment meeting the threshold criteria in the region, altogether yielding a total genomic fraction of 413 945 bp, or approximately 20.6 % of the genome. Here, we use the locations of full (not partial) IS elements as detected by the database IS Finder [48], resulting in 24 located on the chromosome (IS elements and their annotations are listed in Table S7). The positions of 16 of these 24 IS elements overlapped with the 16 CNV hotspot peaks (Fig. 7). To determine the significance of this overlap, the positions of the 24 IS elements were randomly assigned throughout the main chromosome and the number of those elements which fell within the CNV peaks was recorded for each of 1000 iterations. None of the 1000 iterations resulted in an overlap of more than 16.

## Results

### Development of an automated workflow to detect genomic breakpoints in microarray data for archaea

While much is known about the instability of the haloarchaeal megaplasmids [19–21], the stability of the main chromosome remains unclear. To address this question, we developed a computational workflow to detect large-scale genomic variation in existing microarray data for *H. salinarum* (Fig. 1). These data were generated from transcriptomics and ChIP experiments (ChIP-chip, control genomic DNA; Fig. 1a). First, the genome was segmented computationally into various size windows using the DNAcopy algorithm [27] (Fig. 1b). Segments were then filtered by size and significance (see Methods). From the genomic segmentation, we built genome-wide frequency maps of: (a) position-dependent correlated probe intensities in the case of ChIP-chip data; and (b) correlated expression of neighbouring genes for transcriptomics data (Fig. 1c). Frequency was defined as the fraction of microarrays in which a particular genomic segment of a certain size was at least 0.5 standard deviations away from the mean across the entire genome (Fig. 1c, Methods).

Although this segmentation method is well established in applications to human genomic CNV in aCGH data [27], this is the first application to archaeal microarray data. To validate this method in *H. salinarum*, we first investigated whether the segmentation algorithm could detect known functionally related, significantly co-expressed genomic regions in this organism. Transcriptomic data from 1154 microarrays were computationally segmented into size windows of 5–100 kb with significantly correlated gene expression (Fig. 2a, see Methods for segmentation details). These microarrays represent transcriptomic data from 56 *H. salinarum* strains (wild type and mutant) exposed to 72 different experimental conditions (e.g. response to various stressors; see Table S1 for conditions, data accessions and publications). We reasoned that size windows of ~10 kb or less would represent monocistronic transcripts and the majority of polycistronic operons, whereas regions ≥20 kb represent large genomic structural variations such as CNVs. Consistent with this hypothesis, the peak detectable at the 5 kb threshold from the pNRC200 megaplasmid map is significantly enriched for previously identified operons involved in siderophore biosynthesis functions (peak 1, pNRC200 coordinates 170 846–177 665; arCOG enrichment hypergeometric test *P*<0.0123; Fig. 2a, right). Because this operon is subject to strong differential expression during iron fluctuations [47, 49], this peak serves as a proof of concept for application of the DNAcopy segmentation algorithm to archaeal data.

Also as expected, the gas vesicle biogenesis gene cluster (pNRC100 : 16 451–25 376 bp) was differentially expressed in response to growth rate and oxygen tension (log phase and high oxygen conditions represent 10 % of all arrays; low oxygen and stationary phase represent 22 % of arrays; [50]; Fig. 2a, left). These results are consistent with the known function of gas vesicles in maintenance of cell buoyancy during oxygen fluctuation [51]. In a third example consistent with previous results, several genes encoding functions in transposase activity were detected at coordinates pNRC200 : 287 504–331 672, although this enrichment was not significant (Fig. 2a; Peak 2; arCOG enrichment *P*=0.119). As operons are typically co-transcribed in archaea such as *H. salinarum* [52], the recapitulation of these differential expression patterns across the megaplasmids confirms that the DNAcopy segmentation algorithm can be robustly applied to detect co-expressed gene clusters in transcriptomic data.

### Evidence of gene expression changes at large, multi-gene loci across the megaplasmids during stress exposure is consistent with CNVs

To remove individual operons and other large, co-expressed syntenic gene clusters from subsequent analysis and therefore select for those events most probably caused by genomic instability, we next considered only those co-expressed segments 20 kb or larger (Fig. 2b, Table 1). In pNRC100, gene expression is downregulated over the entirety of this megaplasmid in about 5 % of the arrays (Fig. 2b, left). Most of these arrays are from a single large experiment: the long-term tracking of gene expression over time in diurnally entrained cultures [53]. Because gene expression is downregulated in the megaplasmid over the entire length of the time course, regardless of light conditions, it is possible that the observed changes are not due to repression, but rather due to complete loss of the duplicated region of this plasmid in the original culture.

**Table 1.** Statistics regarding numbers of segments detected in microarray data

**Genomic element**	**Total no. of segments**	**No. of significant segments**	**% Arrays with sig. segments**	Mean no. of sig. segments per array*
**Transcriptomics data – 1154 total arrays**				
Chromosome	19 023	535	34.7	1 (1–4)
pNRC100	155	118	9.9	1 (1–2)
pNRC200	222	154	10.1	1 (1–4)
**ChIP-chip data – 48 total arrays**				
Chromosome†	1109	354	87.5	8 (2–18)

*Entries listed as: mean (range). Sig., significant.

†Only the main chromosome was considered in ChIP-chip analysis.

In pNRC200, two regions are frequently coordinately downregulated – one upstream of ~139 kb and one downstream of ~276 kb (Fig. 2b, right). As described above, the region downstream of ~276 kb is enriched for transposase functions, suggesting a source for genomic instability in this region. In contrast, the central, non-duplicated region of pNRC200 is only observed to be coordinately upregulated. Taken together, these results are consistent with the hypothesis that the correlated expression patterns are due to frequent deletion or amplification of DNA on the megaplasmids.

### Megaplasmid copy number amplification results in large-scale coordinated upregulation of gene expression

**Fig. 3.**
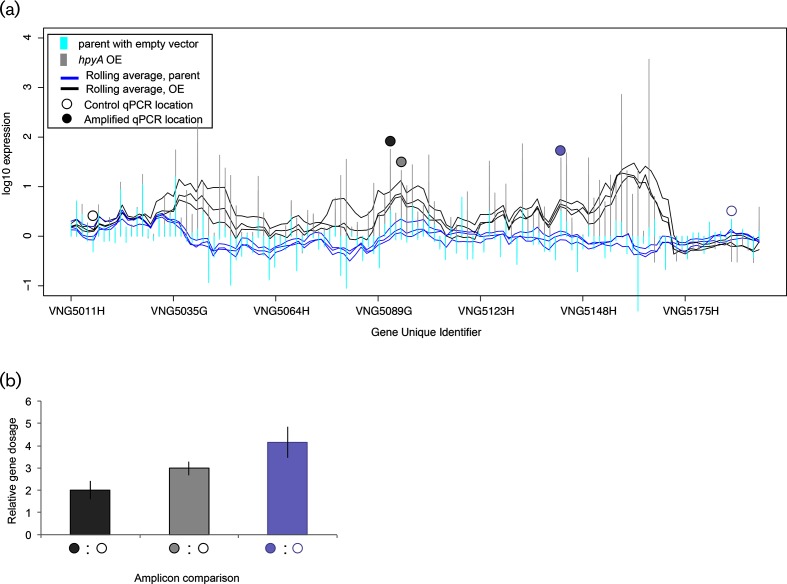
Large-scale genomic amplification influences gene expression. (a) Gene expression changes in mid-logarithmic phase cultures of histone overexpression strain (‘*hpyA* OE’ in legend, grey vertical lines) vs. parent control strain with empty vector (cyan lines) in the megaplasmid pNRC100. Overlaid traces (*hpyA* OE in black, parent in blue) represent the rolling average gene expression level in five-gene windows across the genomic region. Genes are labelled by unique identifier [23] on the *x*-axis. Three biological replicate rolling averages are shown. Gene expression data for this region for stationary phase cultures are shown in Fig. S1. Dots above the vertical lines represent the positions of primers for real-time qPCR of gene dosage, with open circles representing the non-amplified genomic control loci and filled circles depicting the amplified query regions. Primer sequences and genomic coordinates are given in Table S2. (b) Relative gene dosage of genomic loci quantified by qPCR. Dark grey bar shows the ratio of DNA quantity of ORF *VNG5097* [represented by dark grey filled circle in (a)] to non-amplified *VNG5019* [represented by black open circle in (a)]. Light grey bar, ratio of *VNG5102* to *VNG5019*. Light blue bar, ratio of *VNG5148* to *VNG5192*.

To validate that gene expression is directly affected by CNVs, we conducted DNA copy number analysis by real-time qPCR on genomic DNA in the same strain and conditions under which gene expression changes were observed. First, we selected a putative amplification event in the megaplasmid pNRC100 that was detected in the transcriptomics microarray data. These data measured expression in strain KAD102 over the course of the growth curve. This strain overexpresses the *hpyA* gene (unique ID *VNG0134G*; Table S2), which encodes the putative histone-like protein of *H. salinarum* [28]. We observed that pNRC100 of KAD102 contains a 93.5 kb region (pNRC100 coordinates: 38 837–132 357) that is upregulated across both logarithmic phase (Fig. 3a) and stationary phase growth conditions (Fig. S1). In contrast, gene expression in this region remains unchanged relative to the flanking regions in the control strain, KAD101 (Fig. 3a, Fig. S1). This region encompasses approximately half of the genes encoded on megaplasmid pNRC100. qPCR amplicons within this region showed 2- to 4-fold higher DNA copy number than amplicons flanking either side of the upregulated region (Fig. 3b). Although the amplified region of pNRC100 identified here partially overlaps with the previously identified large region of identity between the two megaplasmids (Fig. 2c; region of identity pNRC100 :  1–111 987; [23]), the breakpoints differ, suggesting a novel amplification event. Together, these results suggest that DNA amplification across nearly half of the *H. salinarum* megaplasmid pNRC100 can lead to large-scale, coordinated upregulation of gene expression, which is detectable by microarray. These results are consistent with previous reports of genomic instability on the megaplasmids of *H. salinarum* [19–21] and extend knowledge to include new CNV events and methods of detection.

### Microarray data from transcriptomics and ChIP-chip control hybridizations suggest that CNVs occur on the main chromosome

**Fig. 4.**
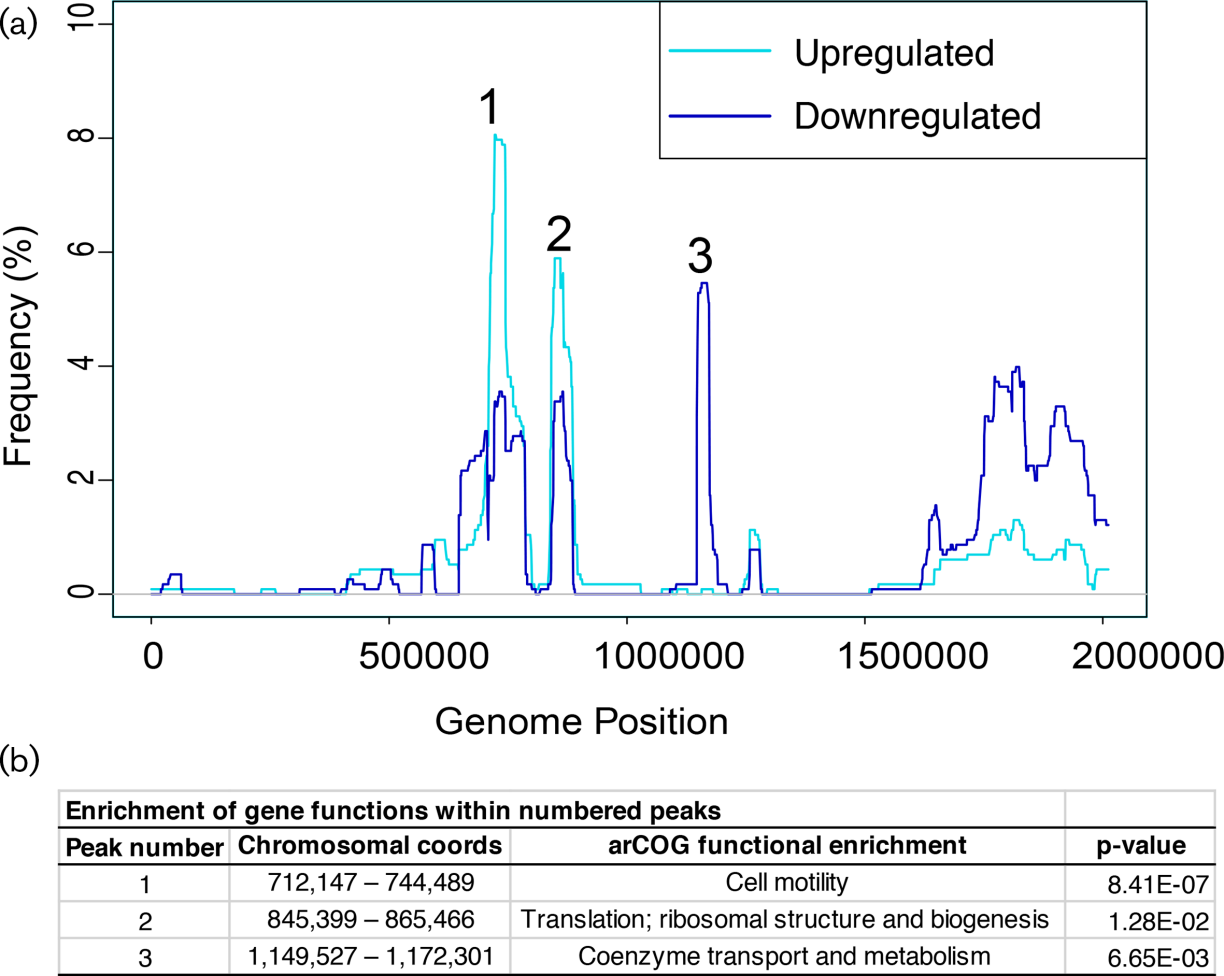
Large syntenic gene clusters are co-expressed. (a) Composite map of significant segment frequency across the chromosome for all 1154 transcriptomics arrays. The frequency of events by chromosomal coordinate in bp is shown for upregulated regions (cyan) or downregulated regions (blue). (b) Significant enrichment of gene functions within peaks numbered in Fig. 4(a). *P*-values indicate the significance of enrichment from the hypergeometric test. Enrichments are also described in the main text.

To gain a genome-wide view, we next investigated the frequency and location of CNVs throughout the main chromosome of *H. salinarum*. In the transcriptomics dataset, the segmentation algorithm detected three major genomic regions on the chromosome ≥20 kb whose expression was at least 1 standard deviation from the mean in ≥5 % of arrays (frequency peaks 1–3, Fig. 4a, Table S4). These peaks include co-expressed gene clusters significantly enriched for gene functions encoding cell motility (peak 1), ribosome biogenesis (peak 2) and cofactor biosynthesis (peak 3; functional enrichment p-values in Fig. 4b). These patterns on the main chromosome could result from CNVs, large clusters of co-regulated genes with common functions, or both (Fig. 4).

In order to differentiate between these possibilities, we used the DNAcopy pipeline (Fig. 1) to analyse scaled raw signal intensities for randomly sheared genomic control DNA from previously published ChIP experiments (see Table S1 for array lists, references and GEO accession numbers; Methods). Data from 48 arrays were analysed, 36 of which were hybridized with DNA from stationary phase cultures, the other 12 from log phase cultures. Represented in this analysis is a total of nine strains, including two to six biological replicates each, plus nine conditions. Segmentation generated a total of 1109 segments on the chromosome across all 48 arrays (Table 1). Because polycistronic signals are not a concern in DNA-based data, we used a 5 kb segment size threshold for analysis of the ChIP data. We reasoned that this would also afford higher-resolution CNV detection. A total of 354 of the chromosomal segments met threshold criteria (≥0.5 standard deviations from mean and ≥5 kb in length; Table 1; Table S5). Of all 48 arrays, 42 contained segments that passed our thresholding criteria, with an average of eight segments per microarray (87.5 % of arrays; range=2–18 per array; Table 1), suggesting frequent CNVs across the chromosome.

**Fig. 5.**
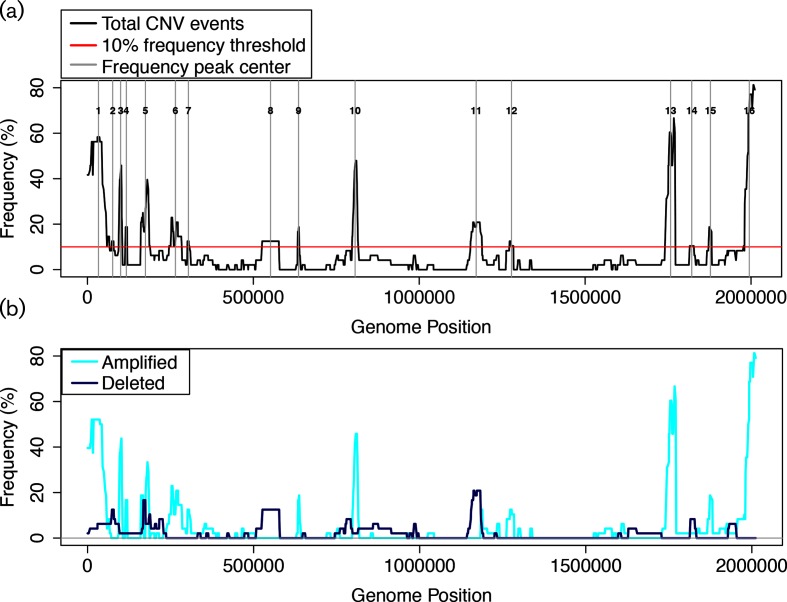
Locations of frequent chromosomal CNVs observed in ChIP input microarray data. (a) Composite map of CNV frequencies across all 48 arrays (black line). Vertical grey lines indicate the centre of CNV regions observed in ≥10 % of arrays. red line indicates the 10% threshold. Numbers on the lines correspond to peak identifiers listed in Table S8, a subset of which are described in the main text. (b) Frequency of events by genomic locus is shown for amplified regions (cyan) or depleted regions (blue).

In order to determine the genomic locations of the most frequently occurring CNVs, we generated a composite map of all significant fragments across all 48 arrays. We detected 16 CNV peaks in the chromosome with an average size of 21.9 kb and a frequency of at least 10 % of arrays (Fig. 5a; Table S8). Separating these peaks by amplification (greater than the mean intensity) vs. depletion (less than the mean) events revealed that DNA amplification is over three-fold more common than depletion in our dataset (Fig. 5b; Table S8). Genes in these 16 CNV regions were significantly enriched for functions in cell wall biogenesis (20 genes, arCOG category M, ‘Cell wall/membrane/envelope biogenesis’) and coenzyme biosynthesis (23 genes, arCOG category H, ‘Coenzyme transport and metabolism’; Table S8). CNV peaks 1 and 10 (Fig. 5a, Table S8) encompass 90 % of cell wall function genes contained within all CNV peaks, including those encoding proteins involved in S-layer glycosylation. The majority of the CNVs in these regions appeared to be amplification events (Fig. 5a). The CNV peak at genetic coordinates Chr: 1 154 809–1 188 524 (peak 11) encompasses 29 genes, of which 17 are predicted to be involved in cobalamin biosynthesis. Genes involved in cobalamin biosynthesis seem to be subject to only genetic depletion.

Consistent with this hypothesis, more than 50 % of peak 11 (~17 kb) overlapped with that of peak 3 in the transcriptomics data, which was also subject only to downregulation (Fig. 4). These data are consistent with the idea that CNVs in the cobalamin biosynthesis gene cluster are associated with changes in gene expression. However, genes in ChIP data peak 1 were not subject to differential expression (compare Fig. 4 to Fig. 5a), suggesting buffering of the S-layer expression phenotype from the effects of CNV. The enrichment of gene functions located in CNV hotspots suggests that some cell phenotypes may change more often than others within the population during evolution.

### Chromosomal deletion events lead to down-regulation of gene expression – validation by RNA-seq and qPCR

**Fig. 6.**
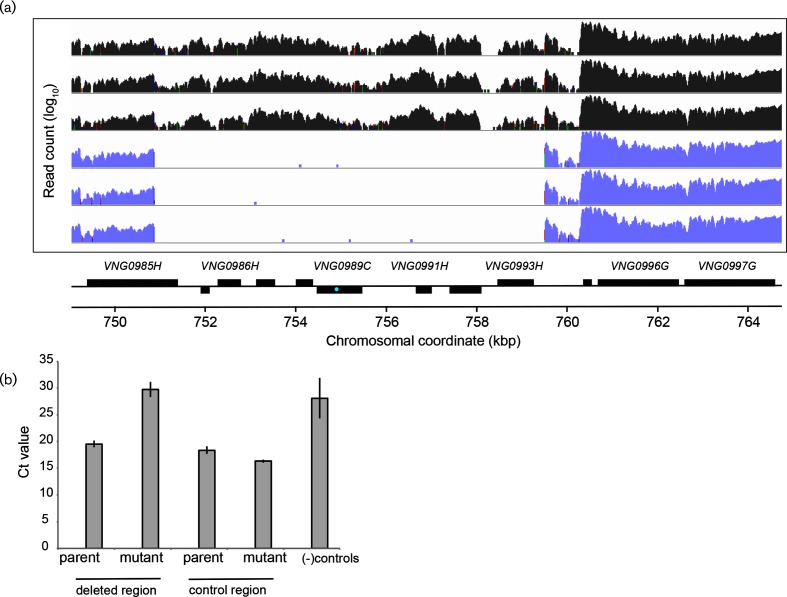
RNA-seq and qPCR validation of deletion events in the *H. salinarum* chromosome. (a) Zoomed genome browser (Integrated Genomics Viewer, [64, 65]) images of sequencing read counts from three RNA-seq biological replicate samples for parent and knockout mutant strains (see Table S2 for strain details). IDs and locations for annotated genes are labelled below the browser image (NCBI Gene database annotation date 30 January 2018). Genes on the forward strand are located above the line, those on the reverse strand below the line. Chromosomal coordinates are indicated by the scale bar. Locations of primers used for qPCR are indicated by the cyan dot within the *VNG0989C* gene. (b) qPCR threshold cycle (*C*_t_) crossing point values of the mutant strain DNA are compared to those of the parent strain and negative controls (water, *E. coli* genomic DNA). Primers amplify the region from 754 459 to 755 481 bp, encompassing the suspected VNG0989C deletion. Bars represent the mean of three biological replicates, each with three technical replicates. Error bars depict the standard deviation of the mean. The difference between parent and mutant strain is significant by two-tailed *t*-test of equal variance (*P*<0.01). Primers are listed in Table S2.

To further test how large-scale instability on the main chromosome of *H. salinarum* affects gene expression, we conducted transcriptomics by next-generation sequencing of RNA (RNA-seq) under mid-logarithmic growth conditions on two strains of *H. salinarum.* These strains are isogenic except for a single gene mutation (∆*ura3* parent vs ∆*hlx2* isogenic derivative; Methods; Table S2). RNA-seq was performed to increase resolution relative to microarray experiments for defining CNV breakpoints. We observed that sequencing reads were not detectable in the ∆*hlx2* strain in a six-gene region of the main chromosome (coordinates 750 868–759 478; genes VNG0986H–VNG0993H; Fig. 6a; Table S6). In contrast, active gene expression was detected in the parent control strain in this region, with read depth varying between 81 and 1112 reads per gene (Fig. 6a; Table S6). This region missing from the mutant strain includes genes of unknown function and a putative phage integrase, VNG0989C. To differentiate whether this difference was due to regulation of gene expression or due to CNV, we conducted qPCR on genomic DNA. Amplicons within this region were detected at significantly higher threshold cycle (*C*_t_) values in the mutant relative to the parent strain (Fig. 6b), indicating a lower concentration of DNA. In contrast, a control locus at chromosomal coordinates 1 296 551–1 296 667 showed similar gene expression levels across control and mutant strains, and had indistinguishable DNA quantity across strains (Tables S2 and S6). Mutant strain DNA quantities from the putative deleted region were indistinguishable from negative controls (no template, water template and *E. coli* DNA template; Fig. 6b), indicating a deletion event had indeed occurred in the genomic region surrounding the integrase gene. However, other than *hlx2* itself, this was the only deletion event detected in this strain across all three biological replicate experiments, suggesting that the chromosome was otherwise stable during the construction of this mutant.

### IS elements are strongly associated with CNV positions throughout the genome of *H. salinarum*

**Fig. 7.**
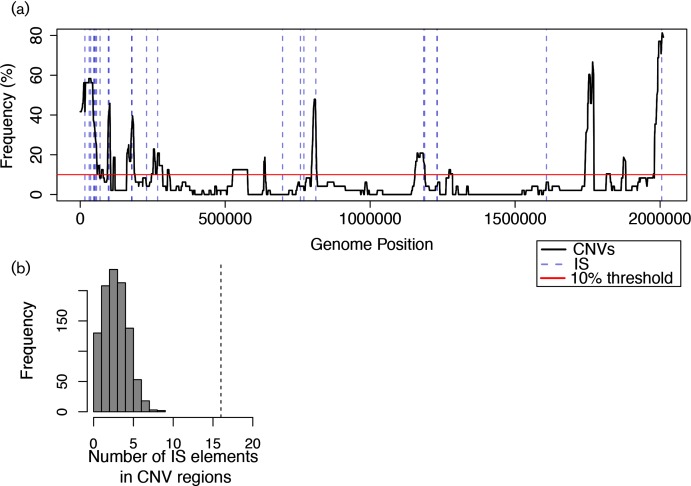
CNVs are significantly associated with IS elements. (a) Location of overlap of all CNVs (black line) with IS elements (dotted grey lines). (b) IS elements fall within the 16 CNV hotspot peak regions (above the red line, see also Fig. 5) more frequently than expected by chance (see Methods for statistical procedure). Dotted line in (b) corresponds to the observed number of IS elements that lie within CNV hotspot peaks. Histogram represents the results of statistical tests (see Methods).

Potentially destabilizing mobile genetic elements such as IS elements are detectable throughout the genome of *H. salinarum* [23, 48]. For example, the amplified region of pNRC100 that we detected here (Fig. 2) is flanked by IS elements (641 bp away from the 5′ end of the 93.5 kb region, and directly demarcating the 3′ end of the region). Such IS elements have previously been shown to be associated with megaplasmid DNA plasticity in *H. salinarum* [15, 54]. In the genomic DNA ChIP data, many CNVs occurred in regions either spanning one or more IS elements or in those regions flanked by IS elements (Fig. 7a; Table S7). Specifically, 16 of the 24 chromosomal IS elements were located within CNV hotspots in the main chromosome (Fig. 7a), an association significantly higher than what would be expected by chance (*P*<0.001, Fig. 7b, Methods). The 5 kb segment size threshold used for ChIP data analysis is approximately twice the size of the largest IS element (~2 kb; [5]), and therefore the segments are not merely indicative of the amplification of individual IS elements themselves. This strong association of CNVs with IS elements is consistent with the hypothesis that mobilization of IS elements or recombination between IS repeats may lead to structural variation throughout the genome. This structural variation changes gene expression at some loci, while other loci remain protected from phenotypic effects (Figs 2, 3, 4 and 6).

## Discussion

Here we have quantitatively assessed genomic plasticity in various strains of a model archaeal species, *H. salinarum*, through the analysis of large transcriptomics and whole-genome DNA microarray data sets. We demonstrate that CNVs are widespread across the genome, but more common and encompassing larger regions on the megaplasmids than on the main chromosome (Figs 2, 4 and 5, Table 1). These results recapitulate reports of frequent genetic rearrangements in the megaplasmids of *H. salinarum* (rates as high as ~10^−2^; [14, 15, 54, 55]). For example, here we observed frequent downregulation of the region downstream of the breakpoint at approximately 276 kb on pNRC200. This region corresponds to region T, a segment flanked by an ISH8 element which was also previously observed as a repositioned segment in strain R1 [21]. Previous reports noted point mutations throughout the main chromosomes of different *R1* and *NRC-1* strains of *H. salinarum*, but did not explore genomic rearrangements on the main chromosome [21]. Here we detected correlated changes in gene expression in regions ≥20 kb across all three genomic elements (Figs 2 and 4). Many, but not all, of these gene expression changes were associated with CNV hotspots (Figs 3, 4, 5 and 6) and possibly caused by IS transpositions (Fig. 7). Therefore, this study expands what is known about the consequences on gene expression of genomic instability across the entire genome of a model archaeon.

Here we show that CNVs are significantly associated with IS elements across the *H. salinarum* chromosome, leading to 16 CNV hotspots (Figs 5 and 7). This result is consistent with previous work in bacteria, as mobile IS elements have been shown to facilitate genomic instability (rearrangement, amplification or deletion) through spurious recombination [1, 4]. Given the recent proliferation of genomics data across strains of bacterial species (e.g. RNA-seq, microarray gene expression data, DNA resequencing data), testing the circular binary segmentation algorithm to determine the effects of genomic structural variation on gene expression in a diversity of organisms is an interesting avenue for future work.

Here we have used the 24 known IS elements listed in the ISFinder database [48] as a conservative estimate of overlap with detectable CNVs. However, in bacteria and archaea, other types of mobile elements can lead to instability [1, 5, 56]. For example, integrases that mobilize elements such as self-replicating plasmids or viruses have been detected in archaeal genomes, including *H. salinarum* NRC-1 [56]. In addition to IS elements, here we also detected integrase genes within two amplified CNV hotspots (*VNG0209H* and *VNG0838G* in peaks 5 and 9, respectively; Figs 5 and 7, Table S8), suggesting an additional potential mechanism for genomic amplification worthy of future study. Recent studies have used comparative genomics to detect up to 80 putative IS elements in the genome of *H. salinarum* [8]. Thus, the contribution of these novel IS elements or other sequences in the ‘mobilome’ to genomic plasticity of *H. salinarum* remains to be determined. Nevertheless, consistent with our observation of the ability of chromosomal IS elements to generate instability in *H. salinarum*, the integration of an entire megaplasmid into the chromosome of *Haloferax volcanii* between repeated ISH18 loci has been observed [57]. Taken together, these data suggest that IS elements play a key role in generating insertions, duplications and deletions in haloarchaeal chromosomes in addition to the megaplasmids.

Here we observed a strong association between CNVs and gene expression change at some loci but not others. For example, deletion events were observed in the cobalamin biosynthesis cluster, which was associated with downregulation of expression (Figs 4 and 5). In contrast, CNVs detected in the cell wall biosynthesis cluster were not associated with significant changes in gene expression (Figs 4 and 5), which could originate from the use of overlapping but not identical strains and growth conditions in transcriptomic and sheared genomic DNA microarray datasets (Table S1). However, CNVs and gene expression were strongly associated in targeted validation experiments at two specific loci in matched strains (Figs 3 and 6) and in other loci in our high-throughput microarray datasets (Figs 4 and 5). In addition, highly polyploid genomes of halophilic archaea could buffer the effects of CNVs on gene expression, where duplications or deletions in these regions may not extend to all copies of the genome [16, 58]. Indeed, heterozygosity may be carried in a haloarchaeal population over several generations if selective pressure is applied [18]. This study therefore raises important questions regarding the relationship between polyploidy, heterozygosity, genomic stability and gene expression in the haloarchaea.

Our routine culturing conditions are designed with minimal serial passaging, which is intended to maintain genomic integrity (Methods). This is reflected in the data, where CNVs on the main chromosome appear minimal in any individual array (i.e. strain) studied here (Table 1). Nevertheless, up to 32 generations of growth from frozen stock to generate sufficient material for RNA or DNA extraction are unavoidable in our hands. Previous work detected widespread rearrangements on the megaplasmids in *H. salinarum* after 34 generations [14]. Plasticity during in-lab culturing has also been observed in other archaeal species. For example, a 124 kb deletion in *Sulfolobus solfataricus* was observed in just one of two biological replicates from the same plate [13]. Given the genomic instability observed in halophiles and other archaea, we expect that excessive serial passaging during in-lab culturing (e.g. routinely maintaining strains by continual re-streaking on plates rather than recovering directly from frozen storage) would compromise strain integrity and lead to the accumulation of genomic rearrangements in strains over time. Concomitant genome resequencing and transcriptomics experiments in cultures grown from the same colony may be a way to clarify whether gene expression changes are due to regulation vs. alterations in genomic structure.

Stress induces IS activity and genome instability. For example, IS element mobility can be induced by long-term incubation of *H. salinarum* plates in the cold [59]. The amplification CNV event validated here was induced merely by introducing an expression plasmid and maintaining via mevinolin selection (Fig. 3, Methods). This suggests that selection used during routine laboratory procedures to maintain strains may result in indirect genetic changes. Consistent with this, the amplified region did not contain the overexpressed gene of interest or genes strongly differentially regulated in the corresponding knock-out mutant of the same gene [28]. We also did not observe CNVs in regions of the genome related to the selection itself (mevinolin resistance and HMG-CoA reductase), suggesting that the CNVs were generated by the activity of endogenous IS elements. Our work is therefore consistent with the hypothesis in the field that stressors introduced during routine laboratory culturing can induce the activity of IS elements [2, 59].

Genomic instability in the halophiles may enable the generation of genetic diversity. These organisms reside in salterns and salt lakes subject to intense solar UV radiation and desiccation/rehydration cycles, which has selected for highly efficient and numerous DNA damage repair mechanisms [17]. For example, the haloarchaea encode homologues from most of the DNA repair pathways found in bacteria and eukaryotes, including base excision repair, nucleotide excision repair, homologous recombination, translesion synthesis and photoreactivation [17, 23]. *H. salinarum* mounts a robust protective response to DNA-damage-inducing stressors, such as UV and gamma radiation, and is capable of rapidly repairing double-stranded DNA breaks (DSBs) in the absence of light [37, 60, 61]. The mutation rate at the single nucleotide level is estimated to be low, which has been confirmed in the related species *Haloferax volcanii* [24]. Large-scale sequence comparison studies that quantified the number of IS elements in more than 1700 bacterial and archaeal genomes showed that the *H. salinarum* genome contains 80 IS elements, a number above the per-genome average across the dataset [8]. Homologous recombination also mediates the integration of DNA through horizontal gene transfer [1] and the exchange of large fractions of the genome during interspecies mating of halophilic archaea [62, 63]. In light of these data, we propose that homologous recombination at IS elements or other short interspersed regions of homology is an important method for generating diversity in *H. salinarum* and potentially other radiation-resistant organisms. Relating particular CNVs to phenotypic consequences, organism fitness and selective pressure therefore poses an important challenge for future work.

## Data bibliography

1. Dulmage KA, Darnell CD, Vreugdenhil A, Schmid AK. Duke digital repository. https://dx.doi.org/10.7924/r4pz54w7h. (2018).
2. Darnell CD, Schmid AK. Gene expression omnibus GSE99730 (2018).
3. Darnell CD, Schmid AK. Sequence read archive SRP108734 (2018).
4. Dulmage KA, Schmid AK. Computer code GitHub repository. https://github.com/amyschmid/Halobacterium_CNV (2018).

## Supplementary Data

Supplementary File 1

Supplementary File 2

Supplementary File 3

Supplementary File 4

Supplementary File 5

Supplementary File 6

Supplementary File 7

Supplementary File 8

Supplementary File 9
