# Supplementary material for: Copy number variation is associated with gene expression change in archaea
Source: Microb Genom. 2018 Aug 24;4(9):e000210. doi: 10.1099/mgen.0.000210 (PMC6202454; doi:10.1099/mgen.0.000210)
Supplement: Supplementary File 9 [file mgen-4-210-s009.pdf]

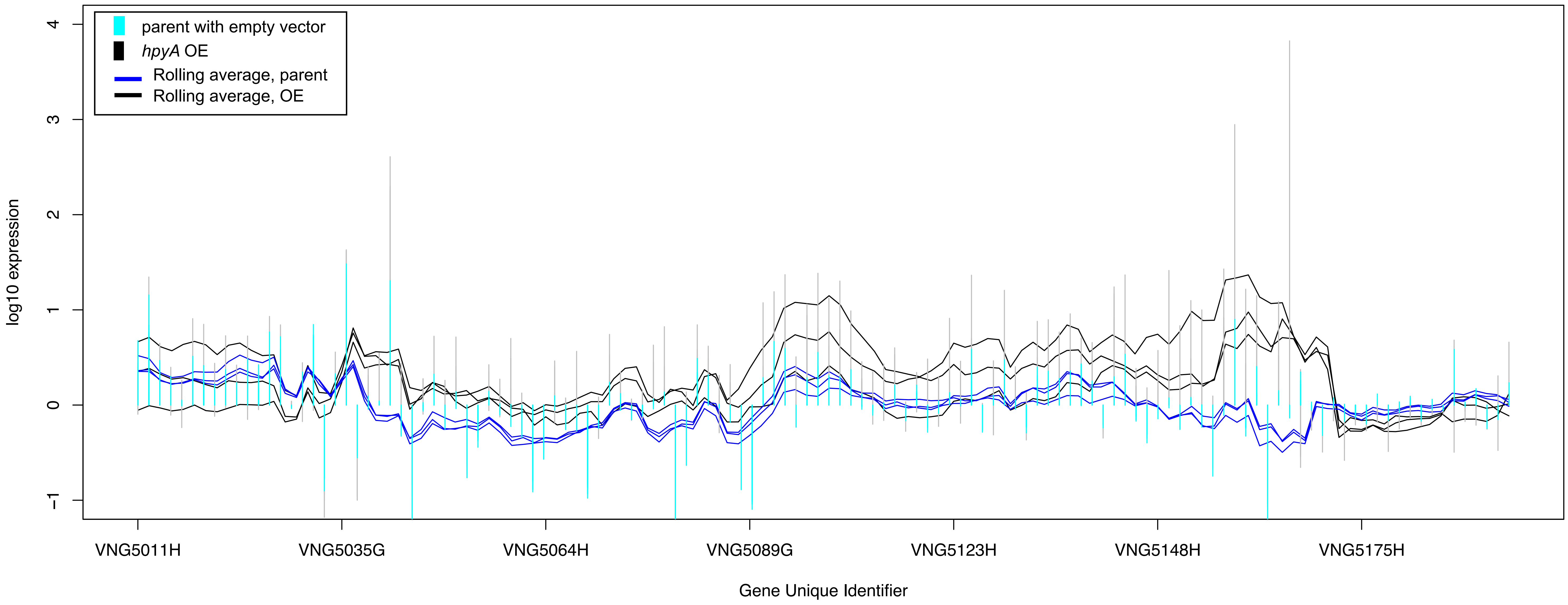

Supplementary Figure S1. Gene expression changes in stationary phase cultures of histone overexpression strain ("hpyA OE" in legend, grey vertical lines) vs. parent control strain with empty vector (cyan lines) in the megaplasmid pNRC100. Overlaid traces (hpyA OE in black, parent in blue) represent the rolling average in 5-gene windows. Three biological replicate rolling averages are shown. Gene expression data for this region for logarithmic phase cultures are shown in main text Figure 3.
